# Chemical induction of leaf senescence and powdery mildew resistance involves ethylene-mediated chlorophyll degradation and ROS metabolism in cucumber

**DOI:** 10.1093/hr/uhac101

**Published:** 2022-05-17

**Authors:** Dingyu Zhang, Shengdong Wu, Ning Li, Jiong Gao, Shihui Liu, Shuai Zhu, Zilin Li, Guodong Ren, Benke Kuai

**Affiliations:** State Key Laboratory of Genetic Engineering and Fudan Center for Genetic Diversity and Designing Agriculture, School of Life Sciences, Fudan University, Shanghai 200438, China; Ministry of Education Key Laboratory for Biodiversity Science and Ecological Engineering, Institute of Biodiversity Science, Fudan University, Shanghai 200438, China; State Key Laboratory of Genetic Engineering and Fudan Center for Genetic Diversity and Designing Agriculture, School of Life Sciences, Fudan University, Shanghai 200438, China; State Key Laboratory of Genetic Engineering and Fudan Center for Genetic Diversity and Designing Agriculture, School of Life Sciences, Fudan University, Shanghai 200438, China; State Key Laboratory of Genetic Engineering and Fudan Center for Genetic Diversity and Designing Agriculture, School of Life Sciences, Fudan University, Shanghai 200438, China; College of Horticulture, Hunan Agricultural University, Changsha 410128, China; State Key Laboratory of Genetic Engineering and Fudan Center for Genetic Diversity and Designing Agriculture, School of Life Sciences, Fudan University, Shanghai 200438, China; State Key Laboratory of Genetic Engineering and Fudan Center for Genetic Diversity and Designing Agriculture, School of Life Sciences, Fudan University, Shanghai 200438, China; State Key Laboratory of Genetic Engineering and Fudan Center for Genetic Diversity and Designing Agriculture, School of Life Sciences, Fudan University, Shanghai 200438, China; Ministry of Education Key Laboratory for Biodiversity Science and Ecological Engineering, Institute of Biodiversity Science, Fudan University, Shanghai 200438, China; State Key Laboratory of Genetic Engineering and Fudan Center for Genetic Diversity and Designing Agriculture, School of Life Sciences, Fudan University, Shanghai 200438, China; Ministry of Education Key Laboratory for Biodiversity Science and Ecological Engineering, Institute of Biodiversity Science, Fudan University, Shanghai 200438, China

## Abstract

Timely initiation of leaf senescence is an integral part of plant development and, importantly, an adaptive strategy by which plants cope with various stresses, e.g. to limit the spread of pathogens. Powdery mildew is a major cucumber disease that promotes the initiation/progression of leaf senescence and reduces leaf photosynthesis, resulting in severe losses of yield and quality. However, how powdery mildew induces leaf senescence and how cucumber plants respond to enhance their resistance remain unclear. Here, with established agrochemical induction and pathogen inoculation systems, we demonstrate that both probenazole (PBZ) and powdery mildew activate ethylene (ET) biosynthesis and signal transduction, consequently promoting leaf senescence and enhancing plant resistance to powdery mildew through CsEIN3 to directly upregulate the expression of *CsCCGs* and *CsRBOHs*. Our analysis convincingly suggests that the regulation of leaf senescence and powdery mildew resistance is interconnected and mediated mainly by ET in cucumber.

## Introduction

As sessile organisms, plants may face diverse biotic and abiotic stresses at any stage of their development. Initiating leaf senescence is an adaptive strategy by which plants deal with long-term stresses, as the physiological and biochemical alterations that occur in senescent leaves contribute to plant fitness. Meanwhile, accelerated nutrient remobilization from senescent leaves promotes the development of young leaves and seeds [1–3]. During leaf senescence, a range of physiological, biochemical, and molecular alterations occur, including accelerated chlorophyll degradation and reactive oxygen species (ROS) metabolism, reduced photosynthetic efficiency, increased membrane ion leakage, and elevated expression of senescence-associated genes (*SAGs*) [2, 3]. Chlorophyll degradation is a prominent event in leaf senescence and is the molecular basis for leaf de-greening [4]. Chlorophyll degradation is catalyzed sequentially by chlorophyll catabolic enzymes (CCEs), including the chlorophyll *b* reductases NON-YELLOW COLORING 1 (NYC1) and NYC1-like (NOL), 7-hydroxymethyl chlorophyll *a* reductase (HCAR), Mg-dechelatase NON-YELLOWINGs/STAY-GREENs (NYEs/SGRs), pheophytinase (PPH), pheophorbide *a* oxygenase (PAO), and red chlorophyll catabolite reductase (RCCR). Expression of the corresponding chlorophyll catabolic genes (*CCGs*) is coordinately regulated by phytohormone signals and external stimuli [4].

ROS, including superoxide anion (O_2_^·−^), hydrogen peroxide (H_2_O_2_), singlet oxygen (^1^O_2_), and hydroxyl radical (HO^·^), become active during leaf senescence [2, 3]. In plants, plasma membrane-localized NAPDH oxidases, also known as respiratory burst oxidase homologs (RBOHs), are responsible for provoking ROS bursts [5]. RBOHs are involved in various plant-pathogen interactions and likely act as the vital regulators leading to two ROS waves (i.e. during the pattern-triggered immunity [PTI] and effector-triggered immunity [ETI] phases) [5, 6]. In addition, the RBOH-mediated ROS burst is also widely involved in various responses to abiotic stresses, including drought, heat, salinity, and wounding [7]. Intriguingly, these stress response processes involving an ROS burst are all accompanied by leaf de-greening. Thus far, the exact molecular relationship between chlorophyll degradation and ROS metabolism remains elusive. Recent studies have shown that magnesium deficiency stress inhibits photosynthetic CO_2_ fixation, concomitantly initiating an ROS burst and enhancing *SGR1/NYE1* expression in rice [8]. Loss of function of NYE1/2, nearly blocking chlorophyll degradation, somehow causes excessive free chlorophyll accumulation in mature seeds, leading to ROS-mediated photo-damage of seeds [9]. Although chlorophyll degradation and ROS metabolism are initiated concurrently in most cases, it is still unclear whether the two processes share a common upstream signal. Some clues point to the involvement of major stress-related phytohormones, e.g. jasmonic acid (JA), salicylic acid (SA), ethylene (ET), and abscisic acid (ABA), various components of whose signaling pathways directly or indirectly regulate the expression of genes encoding major chlorophyll degradation and ROS metabolism enzymes [2, 4, 5, 10, 11].

Accumulating evidence implies that leaf senescence and disease resistance are not two independent processes [10, 12]. When encountering pathogens, leaves rapidly initiate a cascade of immune responses to deal with their invasion and gradually show a de-greening phenotype. In the ETI phase, the pathogen-derived effector-induced hypersensitive reaction (HR) causes local necrosis in leaf tissues, which can be defined as a programmed cellular “suicide” or essentially a quick cellular senescence process to prevent the spread of pathogens. Many senescence-associated regulators are directly or indirectly involved in regulating plant responses to pathogens, e.g. MYC2, NONEXPRESSOR OF PATHOGENESIS-RELATED GENES 1 (NPR1), and ETHYLENE-INSENSITIVE3 (EIN3), as well as a range of NAC and WRKY family transcription factors [10]. Recent studies have also shown that NYE1/SGR1 is directly involved in resistance to anthracnose and downy mildew in cucumber [13, 14]. Notably, all the above regulators act downstream of major phytohormone signaling pathways.

Probenazole (3-allyloxy-1,2-benzisothiazole-1,1-dioxide, PBZ), the active ingredient of the agrochemical Oryzemate, is an effective plant resistance inducer. Like most synthetic plant defense elicitors, PBZ does not possess antimicrobial activities *in vitro* [15, 16]. In *Arabidopsis*, PBZ specifically activates SA biosynthesis/signaling through the ISOCHORISMATE SYNTHASE 1 (ICS1)–NPR1 cascade, inducing systemic acquired resistance (SAR) [17, 18]. PBZ and its metabolic derivative saccharin increase the synthesis of JA and enhance the disease resistance of rice and wheat [19, 20]. Interestingly, both JA- and ET-responsive *cis-*elements mediate the response to PBZ treatment, suggesting that PBZ may act through multiple phytohormone signaling pathways [21]. Powdery mildew caused by *Podosphaera xanthii* is a major disease in cucumber, commonly influencing leaf senescence and heavily affecting yield and nutritional quality [22, 23]. Although the role and mode of action of PBZ in inducing disease resistance and leaf senescence have been investigated extensively in various plant species (including rice, *Arabidopsis*, wheat, maize, and tobacco), its efficacy has not been tested on cucurbitaceous crops [17, 20, 24–26]. Here, we report the unexpected finding that PBZ activates the ET biosynthesis and signaling pathway in cucumber, in contrast to the SA pathway in *Arabidopsis* [17, 27]. The activated ET signaling pathway concomitantly promotes chlorophyll degradation and ROS biosynthesis via a CsEIN3–CsCCGs/CsRBOHs module, thereby initiating cucumber leaf senescence and enhancing resistance to powdery mildew. These findings improve our understanding of the molecular relationship between leaf senescence and disease resistance.

## Results

### PBZ treatment enhances powdery mildew resistance and accelerates the senescence of old leaves in cucumber

**Figure 1:**
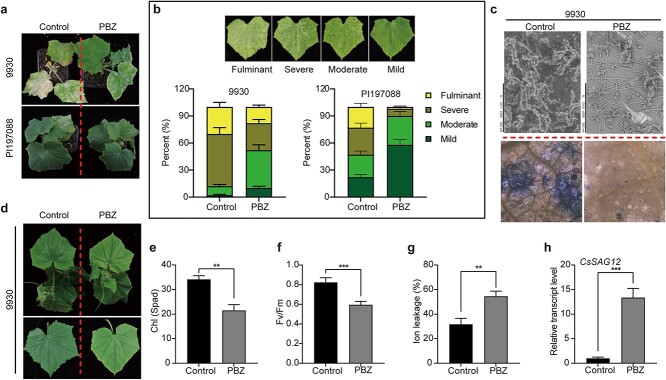
PBZ treatment enhances powdery mildew resistance and accelerates leaf senescence in cucumber. (a) Phenotypes of PBZ- and H_2_O-pretreated cucumber plants at day 14 post powdery mildew inoculation (dpi). Three-true-leaf plants were subjected to PBZ or H_2_O (Control) pretreatment for five days and then inoculated with powdery mildew. (b) A 100% stacked bar chart showing the severity of disease symptoms on infected leaves in PBZ- and H_2_O-treated groups. (c) Upper panel: microscopic examination of hyphae and spores. Lower panel: trypan blue staining of dead cells. The third or fourth leaves from 14-dpi plants were analyzed. (d) Phenotypes of cucumber plants from inbred line 9930 after PBZ treatment. Pictures of whole plants and the first true leaves were taken 14 days after PBZ treatment. (e–h) Chlorophyll content (e), Fv/Fm ratio (f), ion leakage (g), and *CsSAG12* expression (h) in the first and second true leaves of treated plants (d). Data are means ± SD (*n* = 3 biological replicates). ^*^P < 0.05, ^**^P < 0.01, ^***^P < 0.001 (*t*-test).

The plant defense elicitor PBZ induces disease resistance in various plant species [17, 20, 24–26]. We were curious to test whether PBZ could improve powdery mildew resistance in cucumber. Three-true-leaf plants of the powdery mildew–susceptible inbred line 9930, pretreated with PBZ via root drenching for five days, were inoculated with powdery mildew (*Podosphaera xanthii*). Two weeks later, their disease severity was scored according to a four-grade scaling system: mild, moderate, severe, and fulminant. As shown in Figure 1a,b, PBZ pretreatment significantly enhanced resistance to powdery mildew; the proportions of leaves with mild and moderate symptoms were much higher in the PBZ-pretreated group than in the control group. Interestingly, PBZ treatment could further enhance the powdery mildew resistance of the resistant inbred line PI197088. Electron microscopy and trypan blue staining revealed that powdery mildew hyphae/spores were dramatically reduced in PBZ-treated samples, as was cell death (Figure 1c). These results suggest that PBZ effectively enhances powdery mildew resistance in cucumber.

Plant resistance is closely related to physiological and metabolic status [28–30]. We recently reported that PBZ accelerates the senescence process of old leaves in *Arabidopsis* [27], and it was therefore intriguing to know whether PBZ treatment would produce a similar effect in cucumber. As shown in Figure 1d, cucumber plants gradually de-greened from the leaf margin after PBZ treatment. In addition to chlorophyll degradation, we also detected reduced maximum photochemical efficiency of PSII (Fv/Fm), increased ion leakage, and enhanced *CsSAG12* expression in PBZ-treated cucumber leaves (Figure 1e–h)*.* These results demonstrate that PBZ can also accelerate leaf senescence in cucumber.

### PBZ promotes ET biosynthesis and signal transduction in cucumber

**Figure 2:**
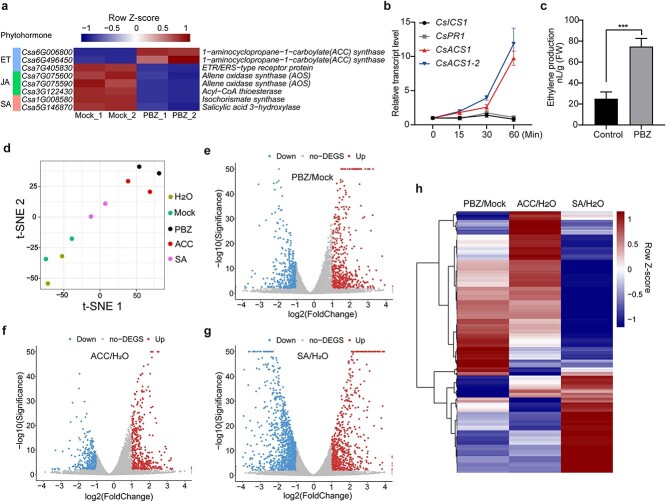
PBZ treatment activates CsACS-mediated ET signal transduction in cucumber. (a) Heatmap of DEGs related to SA, ET, and JA biosynthesis/signaling in 4-hour PBZ-treated cucumber leaves (*n* = 2 biological replicates). (b) Expression time course of *CsACS1*, *CsACS1-2*, *CsICS1*, and *CsPR1* in response to PBZ treatment. Primers used for qPCR are listed in Table S1; data are means ± SD (*n* = 3 biological replicates). (c) ET production in PBZ- and H_2_O-treated cucumber. Data are means ± SD (*n* = 3 biological replicates); ^***^P < 0.001 (*t*-test). (d) *t*-SNE visualization of transcriptomes from different treatments. Tissues were collected after a 30-minute treatment (*n* = 2 biological replicates). Mock treatment (control for PBZ): 0.1% (v/v) acetone solution containing 0.05% (w/v) Tween-20. (e–g) Volcano plots of the DEGs for 30-minute PBZ vs. mock (e), ACC vs. H_2_O (f), and SA vs. H_2_O (g). (h) Heatmap of DEGs in e–g. DEGs from PBZ vs. mock, ACC vs. H_2_O, and SA vs. H_2_O were combined.

In *Arabidopsis*, PBZ induction of SAR and leaf senescence primarily depends on the SA pathway, which involves the upregulation of SA biosynthesis via AtICS1 and signal transduction via AtNPR1 [17, 18, 27]. Surprisingly, a pilot transcriptome analysis revealed downregulation rather than upregulation of *CsICS1* (*Csa1G008580*) after PBZ treatment. Three JA pathway genes were also significantly downregulated (Figure 2a). Notably, two ET biosynthetic genes, *CsACS1* (*Csa6G496450*) and *CsACS1-2* (*Csa6G006800*), were sharply upregulated (Figure 2a). Subsequently, we examined the expression time course of these differentially expressed genes during PBZ treatment by qPCR. As shown in Figure 2b, *CsACS1* and *CsACS1-2* were continuously upregulated during a one-hour PBZ treatment, whereas *CsICS1* and the SA signaling marker gene *CsPR1* were not responsive at all to the PBZ treatment. Consistent with the transcriptome data, JA pathway genes were downregulated during PBZ treatment (Figure S1). We also checked the expression of *CsEIN2* and *CsEIN3*, two core ET signaling pathway component genes, and found that neither of them was induced by PBZ (Figure S1). This result was consistent with the fact that abundance/activity of EIN2 and EIN3 is regulated mainly at the post-translational level [31]. Importantly, ET levels were significantly higher in PBZ-treated samples than in mock-treated controls (Figure 2c). We then concluded that PBZ promotes ET biosynthesis in cucumber.

To further examine the possible involvement of different phytohormone signals in PBZ responsiveness in cucumber, a comparative transcriptome analysis was performed among cucumber leaves treated with PBZ, 1-aminocyclopropane-1-carboxylic acid (ACC, precursor of ET biosynthesis), and SA. The *t*-distributed stochastic neighborhood embedding (*t*-SNE) tool was used to analyze similarities in the transcriptomic changes induced by H_2_O (control for SA and ACC treatment), the mock control (control for PBZ treatment), ACC, SA, and PBZ [32]. As shown in Figure 2d, *t*-SNE separated ACC-/SA-/PBZ-treated transcriptomes from H_2_O-/mock-treated transcriptomes in both dimensions, indicating that the ACC, SA, and PBZ treatments significantly reshaped the transcriptome. Interestingly, PBZ treatment was closer to ACC treatment than to SA treatment (Figure 2d), suggesting that the transcriptomic changes induced by PBZ were more similar to those induced by ACC than by SA. Differentially expressed genes (DEGs) were identified in different treatments, with 497 up- vs. 491 down-, 423 up- vs. 336 down-, and 743 up- vs. 1120 downregulated genes following PBZ, ACC, and SA treatments, respectively (Figure 2e–g). Consistent with the t-SNE results, cluster analysis showed that PBZ-induced DEGs were more similar to ACC-induced DEGs (Figure 2h). Strikingly, an overall trend of opposite changes was seen for the SA-induced DEGs, implying a potential antagonistic effect between PBZ/ET and SA signals (Figure 2h). Taken together, these data suggest that PBZ probably works by activating the ET pathway rather than the SA pathway in cucumber.

### The PBZ-induced ET signal regulates *CsCCG* expression via CsEIN3

**Figure 3:**
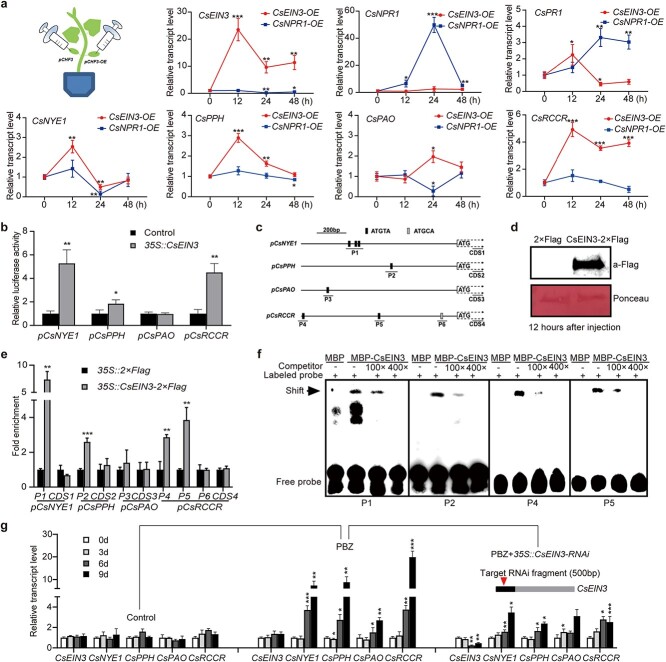
CsEIN3 directly upregulates the expression of *CsCCGs*. (a) Expression time course of *CsEIN3*, *CsNPR1*, *CsCCGs*, and *CsPR1* after overexpression of *CsEIN3* or *CsNPR1* in cucumber cotyledons. Relative transcript levels were calculated as the ratio of *p35S::CsEIN3*-transformed (or *p35S::CsNPR1*-transformed) samples vs. empty vector-transformed samples. (b) Effect of CsEIN3 on the promoter activities of *CsCCGs* by dual-luciferase reporter assay. *pCsCCG::FfLUC* constructs were co-introduced with *p35S::CsEIN3* or the empty vector (negative control) into *Arabidopsis* protoplasts. LUC activities were monitored after 16 hours of culture in the dark at 22°C. (c) Schematic diagrams of EBSs in the promoters of *CsCCGs*. (d) Western blot detection of CsEIN3-2×Flag transiently overexpressed in cucumber cotyledons. Cucumber cotyledons were harvested for analysis at 12 hpi. The *p35S::2×Flag* vector was used as a negative control. (e) ChIP-qPCR analysis of CsEIN3 enrichment in indicated regions (shown in figure 3c) of *CsCCGs*. Chromatin was isolated from *p35S::CsEIN3-2×Flag*- transfected or *p35S::2×Flag-*transfected cotyledons and immunoprecipitated with an anti-Flag antibody followed by qPCR. Enrichment of the target chromatin fragment was normalized to *CsACTIN* and compared with that in the *p35S::2×Flag* control. Coding regions were used as internal controls. (f) CsEIN3 associates with the EBSs in the promoters of the *CsCCGs* in an EMSA assay. (g) Expression time course of *CsEIN3* and *CsCCGs* after RNAi of *CsEIN3* in cucumber cotyledons. Transient RNAi of *CsEIN3* was performed two days after PBZ treatment. Relative transcript levels were calculated as the ratio of *p35S::CsEIN3-RNAi*-transformed vs. empty vector–transformed samples. Primers and probes are listed in Table S1. Data are means ± SD (n = 3 biological replicates). ^***^P < 0.001, ^**^P < 0.01, ^*^P < 0.05 (*t*-test).

EIN3 is a core component of the ET signaling pathway, directly regulating various downstream genes [33–35]. As PBZ activates ET signaling and promotes leaf de-greening in cucumber, we wondered whether CsEIN3 directly regulates *CsCCGs*. We transiently over-expressed *CsEIN3* in cucumber cotyledons, using *CsNPR1* as a control. As shown in Figure 3a, *CsNYE1*, *CsPPH*, and *CsRCCR* showed expression patterns similar to that of *CsEIN3*, reaching their expression peaks around 12 hours post infiltration (hpi). *CsPAO* was upregulated slightly, reaching its expression peak around 24 hpi. By contrast, overexpression of *CsNPR1* did not activate the expression of the *CsCCGs*, although *CsNPR1* and its responsive gene *CsPR1* were significantly upregulated around 24 hpi (Figure 3a). These results demonstrated that transient overexpression of CsEIN3, but not CsNPR1, activated the expression of *CsCCGs* in cucumber cotyledons. To further test whether CsEIN3 directly activates *CsCCGs*, we performed a dual-luciferase assay. A *Firefly Luciferase* gene driven by 1000-bp promoter fragments from individual *CsCCGs* was used as a reporter for CsEIN3-mediated gene activation, with *p35S* promoter-driven *Renilla Luciferase* as an internal control. As shown in Figure 3b, CsEIN3 significantly activated the promoter activity of *CsNYE1*, *CsPPH*, and *CsRCCR* but not that of *CsPAO*. We found two types of EIN3 binding site (EBS) motifs, namely ATGTA and ATGCA, in the activated promoters of the *CsCCGs* (Figure 3c) [36]. Using a transient gene overexpression system in cotyledons (Figure 3d), we further investigated whether CsEIN3 directly binds to these EBSs *in vivo* by chromatin immunoprecipitation followed by qPCR (ChIP-qPCR), with the coding regions as controls. As shown in Figure 3e, CsEIN3-2×Flag robustly bound to P1 (containing three functional EBSs in *pCsNYE1*) with an approximately 7-fold enrichment. Consistent with this result, CsEIN3-2×Flag also strongly bound to P2 of *pCsPPH* and P4 and P5 of *pCsRCCR* but not to P3 of *pCsPAO* (Figure 3e). In addition, we found that CsEIN3-2×Flag did not bind to the ATGCA motif *in vivo* (Figure 3e). Direct interactions between CsEIN3 and EBSs in the promoters of *CsNYE1/CsPPH/CsRCCR* were validated *in vitro* using an electrophoretic mobility shift assay (EMSA) (Figure 3f). To further investigate whether the PBZ-induced expression of *CsCCGs* depends on CsEIN3, cucumber plants were treated with PBZ for two days and then transiently infiltrated with an RNAi construct targeting *CsEIN3*. As shown in Figure 3g, the expression of *CsCCGs* became upregulated around day 6 after PBZ treatment. RNAi of *CsEIN3* significantly reduced *CsEIN3* expression and inhibited PBZ-induced upregulation of *CsCCGs*. These results suggest that PBZ-induced expression of *CsCCGs* largely depends on CsEIN3.

### The PBZ-induced ET signal activates ROS biosynthesis

**Figure 4:**
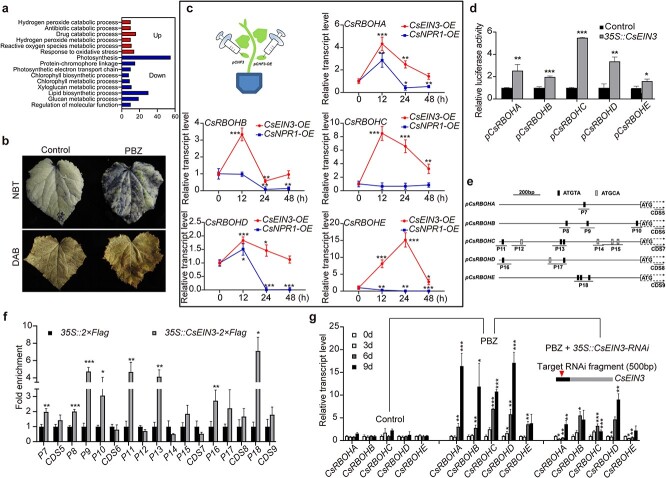
CsEIN3 directly upregulates the expression of *CsRBOHs*. (a) GO analysis of PBZ-regulated DEGs. DEGs from 4-hour PBZ treatment vs. mock treatment were used. The x-axis shows the number of DEGs enriched in the indicated terms. (b) NBT and DAB staining of cucumber leaves after PBZ treatment. PBZ was applied to cucumber via root drenching to a final concentration of 0.5 mM for 14 days, and the first and second true leaves were harvested for the assay. (c) Expression time course of *CsRBOHs* after overexpression of *CsEIN3* in cucumber cotyledons. Overexpression of *CsNPR1* in cotyledons was used as a control. Relative transcript levels were calculated as the ratio of *p35S::CsEIN3*- transformed (or *p35S::CsNPR1*- transformed) samples vs. empty vector–transformed samples. (d) Effect of CsEIN3 on the promoter activities of *CsRBOHs* by dual-luciferase reporter assay. *pCsRBOH::FfLUC* constructs were co-introduced with *p35S::CsEIN3* or the empty vector (negative control) into *Arabidopsis* protoplasts. LUC activities of protoplasts were monitored after 16 hours of culture in the dark at 22°C. (e) Schematic diagrams of EBSs in the promoters of *CsRBOHs*. (f) ChIP-qPCR analysis of CsEIN3 enrichment at the indicated regions (shown in figure 4e) of *CsRBOHs*. Chromatin was isolated from cotyledons transfected with *p35S::CsEIN3-2×Flag* or *p35S::2×Flag* and immunoprecipitated with an anti-Flag antibody, followed by qPCR. Enrichment of the target chromatin fragment was normalized to *CsACTIN* and compared with that in the *p35S::2×Flag* control. Coding regions were used as internal controls. (g) Expression time course of *CsRBOHs* after RNAi of *CsEIN3* in cucumber cotyledons. Transient RNAi of *CsEIN3* was performed two days after PBZ treatment. Relative transcript levels were calculated as the ratio of *p35S::CsEIN3-RNAi*-transformed vs. empty vector–transformed samples. Primers are listed in Table S1. Data are means ± SD (n = 3 biological replicates). ^***^P < 0.001, ^**^P < 0.01, ^*^P < 0.05 (*t*-test).

Gene Ontology (GO) analysis of PBZ-regulated DEGs revealed that downregulated DEGs were mainly enriched in photosynthesis-related pathways, consistent with our observation that PBZ treatment led to leaf senescence of cucumber (Figure 4a; Figure 1d–h). We also found that the upregulated DEGs were mainly concentrated in ROS-related pathways, e.g. hydrogen peroxide catabolic process, antibiotic catabolic process, hydrogen peroxide metabolic process, reactive oxygen species metabolic process, and response to oxidative process (Figure 4a). Using nitroblue tetrazolium (NBT) and diaminobenzidine (DAB) staining, we revealed that the contents of O_2_^·−^ and H_2_O_2_ increased in PBZ-treated cucumber leaves (Figure 4b). These results indicate that PBZ treatment modulates physiological changes involving leaf senescence and ROS biosynthesis in cucumber leaves.

Biosynthesis of apoplastic ROS is mainly catalyzed by NADPH oxidase encoded by *RBOH* [5, 11]. By searching the cucumber (Chinese Long) v3 genome [37], we identified five *CsRBOHs* (*Csa1G038860*, *Csa3G043480*, *Csa4G006160*, *Csa5G002350*, and *Csa5G027600*) that were tentatively named *CsRBOHA*, *CsRBOHB*, *CsRBOHC*, *CsRBOHD*, and *CsRBOHE*. As PBZ treatment enhances the accumulation of ROS in cucumber, we wondered whether these *CsRBOHs* are also regulated by PBZ and CsEIN3. All *CsRBOHs* were significantly upregulated six days after PBZ treatment or 12 hours upon transient overexpression of CsEIN3 (Figure 4c,g). Importantly, RNAi of *CsEIN3* impeded the PBZ-induced upregulation of *CsRBOHs* (Figure 4g). The transcriptional activation of *CsRBOHs* by CsEIN3 was validated by dual-luciferase assay (Figure 4d). ChIP-qPCR further revealed an enrichment of CsEIN3 at EBS regions of *CsRBOH* promoters (Figure 4e,f). As indicated previously (Figure 3e), CsEIN3 significantly associates with ATGTA- but not ATGCA-cored sequence regions (Figure 4e,f). These results indicate that CsEIN3 plays a key regulatory role in PBZ-induced ROS biosynthesis.

### ROS promote the accumulation of *CsCCGs in vivo*

**Figure 5:**
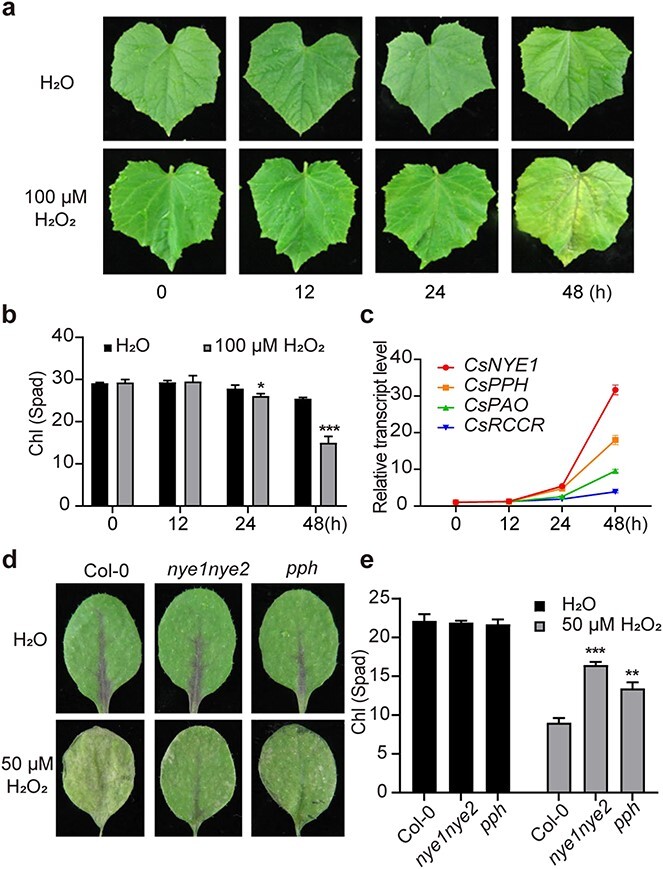
ROS promote leaf de-greening via *CCGs*. (a) Phenotypes of cucumber leaves after H_2_O_2_ or H_2_O treatments. Detached cucumber leaves were treated with 100 μM H_2_O_2_ or H_2_O. Pictures were taken at the indicated time points after treatment. (b) Chlorophyll contents of H_2_O_2_- and H_2_O-treated cucumber leaves. (c) Expression time course of *CsCCGs* in response to H_2_O_2_ treatment in cucumber leaves. (d) *Arabidopsis* leaf phenotypes of the indicated genotypes treated with 50 μM H_2_O_2_ or H_2_O. The third and fourth true leaves of 25-day-old *Arabidopsis* plants were used for treatments, and pictures were taken 48 hours after treatment. (e) Leaf chlorophyll content of the indicated *Arabidopsis* genotypes treated with 50 μM H_2_O_2_ or H_2_O, as described in (d). Primers are listed in Table S1. Data are means ± SD (*n* = 3 biological replicates). ^*^P < 0.05, ^**^P < 0.01, ^***^P < 0.001 (*t*-test).

Chlorophyll degradation is often accompanied by ROS accumulation during leaf senescence. For this reason, we investigated whether ROS affect chlorophyll catabolism. We directly treated detached cucumber leaves with H_2_O_2_, a stable form of ROS *in vivo* [38]. As shown in Figure 5a,b, de-greening of cucumber leaves was significantly accelerated after H_2_O_2_ treatment. A qPCR assay showed that H_2_O_2_ robustly induced the expression of *CsNYE1*, *CsPPH*, *CsPAO*, and *CsRCCR* (Figure 5c). Using *Arabidopsis* mutants of *AtCCGs* and AtCCE antibodies, we found that H_2_O_2_ treatment significantly promoted the accumulation of AtNYE1 and AtPPH in *Arabidopsis* leaves. Dysfunction of either AtNYE1/2 or AtPPH significantly blocked H_2_O_2_-triggered chlorophyll degradation, indicating that ROS-induced leaf de-greening depends heavily on CCEs (Figure 5d,e; Figure S2).

### Involvement of ET-accelerated ROS biosynthesis in defense against powdery mildew in cucumber

**Figure 6:**
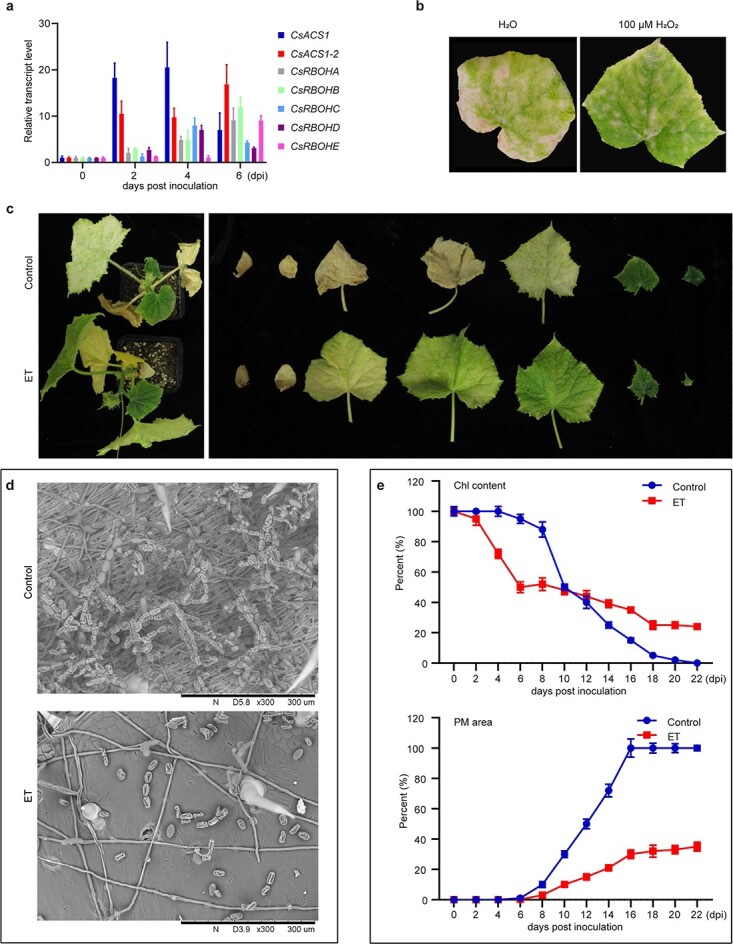
ET-accelerated ROS biosynthesis promotes powdery mildew resistance in cucumber. (a) Expression time course of *CsACSs* and *CsRBOHs* after powdery mildew inoculation in cucumber leaves. Relative transcript levels were calculated as the ratio of powdery mildew–treated vs. 0.01% Tween-20-treated (control) cucumber leaves. Data are means ± SD (*n* = 3 biological replicates). Primers used for qPCR are listed in Table S1. (b) Phenotypes of powdery mildew–inoculated cucumber leaves after H_2_O_2_ and H_2_O treatments. Powdery mildew–inoculated attached leaves were treated with 100 μM H_2_O_2_ or H_2_O once a day. The leaves were detached for photography at 22 dpi. (c) Phenotypes of ET-treated and control cucumber plants and their leaves at 22 dpi. (d) Microscopic observations of the hyphae and spores in the third or fourth leaf of ET-treated and control cucumber plants. (e) Time course of changes in Chl content and powdery mildew area in the ET-treated or control cucumber plants after powdery mildew inoculation. The first and second true leaves of cucumber plants were used for Chl content and powdery mildew (PM) area measurement. Data are means ± SD (*n* = 3 biological replicates). The experiment was repeated at least four times with similar results.

ET and ROS play crucial roles in plant immune responses [36, 39, 40]. To explore whether ET and ROS mediate powdery mildew resistance in cucumber, we first monitored ET and ROS biosynthetic genes at different time points after powdery mildew inoculation. Notably, the PBZ-induced genes *CsACS1* and *CsACS1-2* were sharply upregulated at 2 dpi, whereas *CsRBOHs* were upregulated later, at 4 dpi or 6 dpi (Figure 6a), indicating that ET biosynthesis occurs prior to ROS accumulation during powdery mildew infection in cucumber. Treatment of powdery mildew–inoculated plants with H_2_O_2_ inhibited the growth of the powdery mildew pathogen (Figure 6b). We next treated powdery mildew–inoculated cucumber plants with ET for six days and then cultured them under normal conditions. As shown in Figure 6c,d, ET treatment significantly improved powdery mildew resistance in cucumber. As chlorophyll content could be measured nondestructively by a SPAD-plus meter, we monitored the rate of chlorophyll degradation as a reflection of ROS status. The disease scar area on the ET-treated leaves was significantly reduced compared with that on the control leaves, especially at the beginning of the powdery mildew outbreak (Figure 6e). These results demonstrate that ET-accelerated ROS biosynthesis may significantly improve cucumber resistance to powdery mildew.

## Discussion

### ET biosynthesis/signal transduction mediates PBZ-initiated molecular and physiological alterations in cucumber

Synthetic plant defense elicitors have been widely used in agriculture to improve plant resistance to biotic and abiotic stresses. However, the underlying molecular mechanisms remain elusive [15]. It has been shown that PBZ acts via the mediation of phytohormone biosynthesis and signal transduction to induce disease resistance and leaf senescence [15, 27]. In *Arabidopsis*, SAR is induced almost exclusively by SA biosynthesis and signal transduction, as PBZ-induced *AtPR1* expression was deficient in *NahG* (which encodes a salicylate hydroxylase that converts SA to catechol) transgenic plants and the SA signaling mutant *npr1*, but not in ET or JA signaling mutants [17, 27]. Previously, we revealed that PBZ-mediated SA biosynthesis depends on *ICS1* in *Arabidopsis* [18]. Surprisingly, *CsICS1* expression was not induced by PBZ in cucumber. By contrast, *CsACSs* but not *AtACSs* were sharply upregulated by PBZ (Figure 2a,b, Figure S3). Comparative transcriptome analysis further revealed that the PBZ-triggered global transcriptome changes in cucumber were similar to those triggered by ET rather than by SA (Figure 2). Consistent with the molecular alterations, PBZ treatment also leads to physiological changes similar to those activated by ET, e.g. accelerated chlorophyll degradation and ROS biosynthesis (Figure 1d,e; Figure 4b). Furthermore, in cucumber, ET- or PBZ- mediated signaling seems to be antagonistic to that mediated by SA, as most DEGs between PBZ/ET- and SA-treated transcriptomes showed opposite changes in expression (Figure 2h). Notably, *CsICS1* was significantly downregulated after a long-term treatment with PBZ (Figure 2a). This was not surprising, as the key downstream component EIN3 of the ET signaling pathway was reported to directly repress the expression of *ICS1* in *Arabidopsis* [41]. These observations suggest that although phytohormone biosynthesis and signaling induced by the synthetic compound PBZ have species specificity, at least between cucumber and *Arabidopsis*, the antagonistic relationship between endogenous ET and SA signaling is still conserved between the two species.

### CsEIN3 plays a crucial role in the regulation of chlorophyll and ROS metabolism in cucumber

In the ET signaling pathway, the core component EIN3 acts as a crucial downstream regulator, directly regulating diverse functional genes, including those involved in chlorophyll and ROS metabolism [4, 36, 42, 43]. In cucumber, CsEIN3 directly binds to the ATGTA motif in the promoters of *CsNYE1*, *CsPPH*, and *CsRCCR*, upregulating their expression. However, we did not detect a regulatory role for CsEIN3 in the expression of *CsNYC1* or *CsPAO*, two other major *CCGs*, a result that differs from our findings in *Arabidopsis* (Figure 3; Figure S4), i.e. AtEIN3 directly upregulates *AtNYC1*, *AtNYE1*, and *AtPAO* [43]. This less conserved regulatory relationship between EIN3 and *CCGs* in cucumber and *Arabidopsis* may be due to functional redundancy of EIN3 and EILs.

Apart from chlorophyll degradation, we also found that PBZ promotes ROS production, and the ROS-generating enzyme genes *CsRBOHA/B/C/D/E* are the targets of CsEIN3. In rice, OsEIL1, the closest homolog of EIN3, directly upregulates *OsRBOHs* in defending rice against *Magnaporthe oryzae* [36]. In *Arabidopsis*, stabilized AtEIN3 directly upregulates the expression of *AtPODs*, contributing to ROS scavenging and improving salinity tolerance [42]. In cucumber, the two regulatory pathways may co-exist: CsEIN3 overexpression induced upregulation of both *CsPODs* and *CsRBOHs*, and upregulation of *CsPODs* lagged behind that of *CsRBOHs* (Figure 4c; Figure S5)*.* These observations suggest that CsEIN3 initially induces ROS production via CsRBOHs and then scavenges over-produced ROS via CsPODs, which may be required for maintaining a dynamic but balanced change. SA may also regulate ROS metabolism via CsNPR1, as CsNPR1 overexpression significantly suppressed expression of most *CsPODs*, implying that SA suppresses the ROS scavenging pathway to regulate ROS metabolism (Figure S5). The differential regulatory effects of CsEIN3 and CsNPR1 on ROS metabolism may explain, to a certain extent, why ET can induce a moderate leaf senescence phenotype but SA induces severe programmed necrosis in some plants [3, 44].

### ROS may act as positive feedback signals to regulate chlorophyll degradation

An intriguing observation is that chlorophyll degradation and ROS accumulation generally occur concomitantly during natural leaf senescence and pathogen-incurred de-greening. In this study, we found that, apart from ET signaling, ROS can also positively regulate the transcription and protein accumulation of *CCGs* (Figure 5; Figure S2). These results suggest that ROS can directly or indirectly stimulate the expression of *CCGs*. ROS, especially upon reaching a certain concentration, may act as a retrograde signal, regulating gene expression in the nucleus [11]. An interesting finding is that endoplasmic reticulum (ER)-localized NAC transcription factors, e.g. ANAC13 and ANAC17, can directly sense ROS as a retrograde signal to influence nuclear gene expression [11]. NAC transcription factors are the most important regulators that directly upregulate the expression of *CCGs* during leaf senescence induced by ET and other factors [2, 4, 43]. Therefore, senescence-associated NAC transcription factors may play key roles in sensing ROS signals to upregulate *CCGs*, although more details remain to be elucidated.

### PBZ/ET-initiated ROS/chlorophyll metabolism contributes to the enhancement of powdery mildew resistance in cucumber

PBZ improves disease resistance in crops such as maize, rice, and wheat [20, 24, 26]. However, to the best of our knowledge, there have been no reports on the application of PBZ in cucumber. Powdery mildew is a major disease in cucumber, causing significant losses of yield and harvest quality worldwide [22, 23]. As a fungus, *Podosphaera xanthii* (the causal agent of powdery mildew) spreads via its airborne spores, making it difficult to control. To date, there is no specifically effective fungicide. We found that cucumber resistance to powdery mildew was significantly improved by exogenously applied PBZ (Figure 1a–c). Large-scale verification further confirmed that PBZ induces powdery mildew resistance in a variety of cultivars (Figure S6). PBZ is environmentally friendly and, in particular, not antimicrobial [16, 45], which may preclude the development of fungicide resistance, and our findings may therefore help to develop a new strategy for coping with cucumber powdery mildew.

The role of ET in modulating plant resistance to biotic stresses has been widely reported [39], but how ET signal transduction eventually contributes to pathogen resistance remains poorly understood and sometimes even controversial [41, 46]. It is generally believed that ET is responsible for activation of plant defense against necrotrophic pathogens and SA for biotrophic and hemibiotrophic pathogens [47, 48]. However, this view may be oversimplified, as plant defense against hemibiotrophic pathogens such as *Magnaporthe oryzae* can also be activated by ET [36]. A recent report also showed that ET improves einkorn wheat resistance to the biotrophic *Blumeria graminis* that causes powdery mildew [49]. In this study, we found that either PBZ or ET treatment was sufficient to enhance the resistance of cucumber to biotrophic *Podosphaera xanthii*, the causal pathogen of powdery mildew (Figure 1a–c; Figure 6). Coupling ROS metabolism with leaf de-greening may be crucial for explaining ET- or PBZ- induced powdery mildew resistance, as disease resistance is closely related to alterations in physiological status [28–30]. Leaf senescence and plant immune response are physiologically and molecularly interconnected, as reflected in the following observations: first, all the major senescence regulatory hormones (e.g. SA, JA, and ET) take part in regulating plant resistance to pathogens; second, almost all plant immune regulators modulate the leaf senescence process; finally, ROS production is a core event in both plant immune response and leaf senescence [2, 5, 10]. ROS produced by *RBOH*-encoded NADPH oxidases play a critical role in plant immune responses [5, 11]. In this study, PBZ- or ET-induced powdery mildew resistance was closely related to ROS biosynthesis, suggesting a crucial role for ROS biosynthesis in the control of cucumber powdery mildew (Figure 4; Figure 6).

In conclusion, by taking advantage of PBZ-induced chlorophyll degradation and ROS biosynthesis, we demonstrated that PBZ activates phytohormone signals in a species-specific manner, at least between cucumber and *Arabidopsis*. Further analyses revealed that CsEIN3 regulates both chlorophyll degradation and ROS biosynthesis by directly upregulating *CsCCGs* and *CsRBOHs* in PBZ/ET-promoted physiological alterations, helping to explain why pathogen inoculation always causes leaf de-greening. In addition, our results suggest that the CsACSs–CsEIN3–CsRBOHs/CsCCGs signaling cascade contributes to cucumber resistance to the biotrophic disease powdery mildew. In the future, it will be worth constructing and analyzing more ET-related genetic materials of cucumber, which would provide more reliable genetic data for elucidating the mechanism of ET-induced resistance in cucumber.

## Materials and methods

### Plant materials and growth conditions

*Cucumis sativus* L. ecotype Chinese Long inbred line 9930 was used for most experiments, whereas the inbred line PI197088 was used as a powdery mildew resistant control [50]. *Arabidopsis nye1nye2* and *pph* mutants were generated as described previously [51, 52]. Arabidopsis plants were grown in soil under a long-day photoperiod (16-hour light/8-hour dark) with approximately 120 μmol m^−2^ s^−1^ light intensity. Cucumber plants were grown under the same conditions as *Arabidopsis* up to the two-true-leaf stage and then transferred to a greenhouse at the Jiangwan campus of Fudan University in Shanghai (31.34°N, 121.50°E).

### Pathogen inoculation

Conidia of powdery mildew (*Podosphaera xanthii*) were pre-cultured and maintained on living cucumber plants growing in the greenhouse. For the disease resistance test, spores were washed down and re-suspended in a water solution containing 0.01% Tween-20. The solution was then filtered through gauze, and the spore concentration was adjusted to 10^6^ mL^−1^. Cucumber plants were inoculated with the spore solution by spraying evenly on their surface, and plants sprayed with water containing 0.01% Tween-20 served as the controls. For scanning electron microscopy examination of pathogens, infected leaf tissues were examined with an SU8010 microscope (Hitachi).

### Chemical treatments

For PBZ treatment of living plants, cucumber plants were treated with 0.5 mM PBZ solution via root drenching [27]. For H_2_O_2_ treatment of inoculated living plants, the leaves were sprayed gently with 100 μM H_2_O_2_ solution to form a layer of mist on their surface (to avoid disturbing inoculated pathogens). For ET treatment of living plants, cucumber plants were placed in a sealed glass desiccator in which ET was produced by mixing 5 mM Na_2_HPO_3_ with 0.5 M ethephon stock (1000×), reaching a final concentration of 100 μL/L [43].

For H_2_O_2_ treatment of excised leaves, detached *Arabidopsis* and cucumber leaves were floated on H_2_O_2_ solution (50 μM for *Arabidopsis* and 100 μM for cucumber). For ACC/SA treatment, detached cucumber leaves were floated on 200 μM ACC or SA solution. Excised cucumber leaves were treated with PBZ as previously described with minor modifications [53]. In brief, detached cucumber leaves were floated on PBZ solution (for 1 L PBZ solution, 100 mg PBZ was dissolved in 1 ml acetone, and the resultant solution was then added to 1 L deionized water containing 0.05% [w/v] Tween-20) in Petri dishes, and a 0.1% (v/v) acetone solution containing 0.05% (w/v) Tween-20 was used for the mock treatment.

### Measurement of Chl contents

Chl contents of *Arabidopsis* and cucumber leaves were determined using a SPAD-502 PLUS chlorophyll meter.

### Measurement of Fv/Fm ratios

Maximal photochemical efficiencies of PSII in cucumber leaves (Fv/Fm) were measured with an LI-6400 system (LI-COR) according to the manufacturer’s instructions.

### Measurement of ion leakage

The conductivity of detached cucumber leaves was measured with a digital conductivity meter (Waterproof ECTestr11+ Multi-Range Tester). In brief, the leaves were immersed in deionized water and shaken gently in a 25°C water bath for 60 minutes, and the conductivity values of the resulting solutions were measured (C1). Samples were then boiled for 15 minutes, and the conductivity values of the solutions were measured after cooling (C2). The membrane ion leakage was calculated as the ratio of C1 to C2.

### Measurement of cell death

A trypan blue staining assay was used to determine the severity of cell death in cucumber leaves. In brief, leaf tissues were immersed in trypan blue staining solution [0.25 mg/ml trypan blue, 20% (v/v) lactic acid, 25% (v/v) glycerin, 25% (v/v) saturated phenol] for 4 hours at room temperature and then transferred to water with gentle shaking to de-stain. After that, the leaf tissues were examined under a microscope.

### Measurement of ET production

To quantify ET production, cucumber plants were pretreated with PBZ via root drenching for seven days, with water-treated plants as controls. The first and second true leaves were detached, weighed, and incubated in 40-mL airtight glass bottles for 24 hours to accumulate ET. ET production was measured with the ETD300 ethylene detector system following the manufacturer’s instructions.

### Measurement of ROS

For H_2_O_2_ measurement, cucumber leaves were immersed in 1 mg/ml DAB staining solution (pH 3.8) and placed under a vacuum in the dark for 4 hours. Afterward, cucumber leaves were boiled in 95% ethanol solution to destain before being photographed.

For O_2_^·−^ measurement, cucumber leaves were immersed in NBT staining solution (0.5 mg/ml in phosphate buffer [pH 7.5]) and placed under a vacuum in the dark for 1 hour. Leaves were boiled in 95% ethanol solution to destain before being photographed.

### Measurement of POD activities

POD activities of cucumber leaves were determined with a POD activity detection kit (Comin, Item No. POD-2-Y) according to the manufacturer’s instructions.

### Western blot analysis

Fresh tissues were homogenized in liquid nitrogen and extracted using extraction buffer as described previously [9]. The extraction mixture was denatured at 90°C for 10 minutes and centrifuged at 10 000 rpm for 5 minutes. The supernatant was collected for immunoblot analysis. Proteins were separated via SDS–polyacrylamide gel electrophoresis (PAGE) on a 10% polyacrylamide gel. Target proteins were detected using anti-FLAG (Agrisera), anti-NYE1 (Shanghai Ango Biotechnology), or anti-PPH antibodies (Shanghai Ango Biotechnology).

### RNA extraction and RT-qPCR

Leaf samples were homogenized in liquid nitrogen, and total RNA was extracted using RNAiso Plus (Takara). PrimeScript RT Master Mix (Takara) was used to synthesize cDNA from extracted RNA. Quantitative PCR was performed on a CFX Connect Real-Time PCR Detection System (Bio-Rad) with a TB Green Premix Ex Taq II (Tli RNaseH Plus) kit (Takara). Primers for qPCR are listed in Table S1. The reaction procedure for qPCR was as follows: 95°C for 3 minutes, followed by 40 cycles of 95°C for 15 seconds, 56°C for 30 seconds, and 72°C for 30 seconds.

### RNA-seq and data analysis

Total RNA extraction, library construction, and RNA-sequencing (Illumina NovaSeq 6000/HiSeq X Ten platform) were conducted by Novogene Bioinformatics Institute and Genergy Biotech Co. (Shanghai). RNA-seq data were deposited in the NCBI Sequence Read Archive (SRA) under PRJNA771246. The cucumber reference genome and annotation files were obtained from http://cucurbitgenomics.org/organism/2 [54]. RNA-seq analysis was performed with the DESeq2 R package. DEGs were defined based on cut-off values of log_2_(fold change) ≥ 1 (for 30-minute PBZ/ACC/SA/H_2_O/Mock treatments) or log_2_(fold change) ≥ 2 (for 4-hour PBZ/Mock treatments) and *P* < 0.01. GO over-representation, t-SNE analysis, and heatmap construction were performed using the clusterProfiler, Rtsne, and pheatmap R packages.

### Transient expression assays

The indicated genes were cloned into the p*CHF3* vector (for overexpression) and the *pro35S-Intron* vector (re-designed based on *pMeioDMC1-Intron*, for RNAi) [55], which were then introduced into the *Agrobacterium tumefaciens* GV3101 strain by the freeze–thaw method. *A. tumefaciens* were then cultivated in YEB medium, re-suspended in infiltration medium (0.5% sucrose, 10 mM MES [pH 5.6], 10 mM MgCl_2_, 200 μM acetosyringone, and 40 μl/L Silwet L-77) to a final OD_600_ value of 0.3, and placed under light for 2 hours. The activated *A. tumefaciens* were infiltrated into cucumber cotyledons using a blunt-ended syringe. Cucumber plants were placed under constant light for protein expression, and cotyledons were harvested at the indicated time points.

### Chromatin immunoprecipitation assay

CsEIN3-2×Flag was transiently expressed in cucumber cotyledons, and its expression was confirmed by western blotting. Samples were harvested and cross-linked with 1% formaldehyde under vacuum for 20 minutes and neutralized by adding glycine to 125 mM (final concentration) for an additional 10 minutes. Nuclei were isolated and sonicated, and chromatin immunoprecipitation was performed using an EpiQuik Plant ChIP Kit (Epigentek) with an anti-Flag antibody (Agrisera). After reverse cross-linking, associated DNA was collected using a ChIP DNA Clean & Concentrator kit (Zymo Research). Primers for qPCR are listed in Table S1.

### Electrophoretic mobility shift assay (EMSA)

MBP and MBP-fused CsEIN3 (in the p*MAL-c5g* vector) were expressed in *Escherichia coli* Rosetta (DE3). Proteins were purified with Anti-MBP Magnetic Beads (NEB). EMSA was performed using a LightShift EMSA Optimization and Control Kit (ThermoFisher Scientific) with unlabeled probes as competitors. The 15-μL reaction mixture contained 1.5 μL 10× binding buffer, 0.75 μg Poly (dI·dC), indicated quantities of unlabeled competitive oligonucleotides, 2 μg of target proteins, and 1 μL of 50 fmol biotin-labeled annealed oligonucleotides. The reaction mixture was incubated for 20 minutes at 25°C, electrophoresed on a polyacrylamide gel, transferred to a positively charged nylon membrane (Amersham Biosciences), and UV cross-linked in a CL-1000 Ultraviolet Crosslinker. Biotin signals were detected with the Chemiluminescent Nucleic Acid Detection Module (ThermoFisher Scientific) and visualized with a chemiluminescence image analysis system (Tanon 5200).

### Dual-luciferase reporter assays

*Arabidopsis* protoplasts prepared from the rosette leaves of 3-week-old Col-0 plants were used for dual-luciferase assays. p*GreenII 0800-LUC* plasmids harboring the indicated promoters were co-transformed with *p35S::CsEIN3* constructs (cloned into p*CHF3*) into *Arabidopsis* protoplasts by the polyethylene glycol (PEG)-mediated transformation method. Firefly-Renilla luciferase activities were monitored with the Dual-Luciferase Reporter Assay System (Promega) on a Synergy 2 Multi-Mode Microplate Reader (Bio-Tek).

## Supplementary Material

Web_Material_uhac101

## Data Availability

The authors confirm that the data supporting the results of this study are available in the article and supplementary materials. The raw RNA-seq data have been deposited at the NCBI Sequence Read Archive (SRA) under BioProject accession number PRJNA771246.
