# Supplementary material for: Chemical induction of leaf senescence and powdery mildew resistance involves ethylene-mediated chlorophyll degradation and ROS metabolism in cucumber
Source: Hortic Res. 2022 May 17;9:uhac101. doi: 10.1093/hr/uhac101 (PMC9250653; doi:10.1093/hr/uhac101)
Supplement: Web_Material_uhac101 [file web_material_uhac101.zip › Supplementary data.docx]

**Supplementary data**


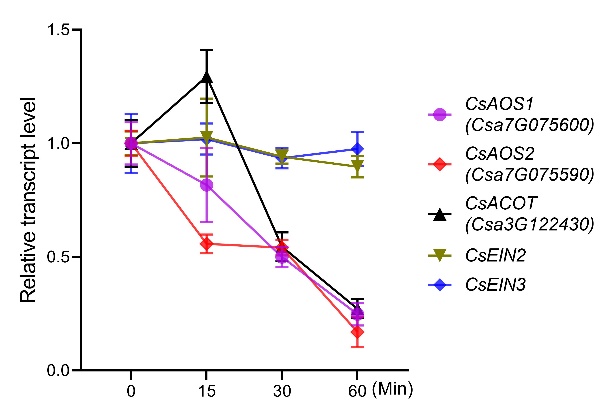


**Figure S1. Time-course expression patterns of JA-** **and ET-related genes in response to PBZ treatment in cucumber leaves.**

Samples were the same as in Figure 2b. *CsACTIN* (*Csa6G484600*) was used as an internal control and the expression level of each gene at time 0 was set to 1. Data are means ± SD (*n* = 3 biological replicates). Primers used for qPCR are listed in Table S1.


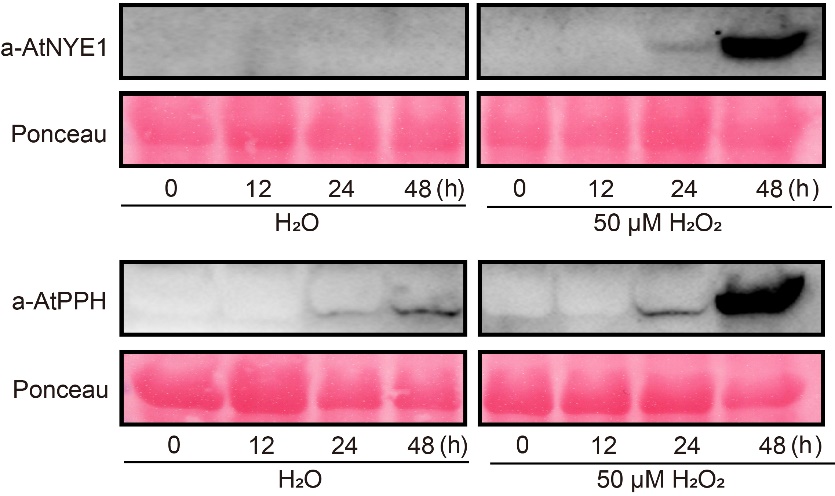


**Figure S2. Time-course protein accumulations of AtNYE1 and AtPPH in response to H_2_O_2_ treatment in Arabidopsis leaves.**

The third and fourth true leaves of 25-day-old *Arabidopsis* plants were used for H_2_O_2_ treatment. H_2_O treatment was used as negative control.


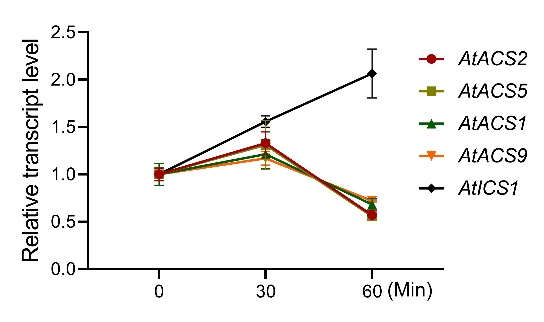


**Figure S3. Time-course expression patterns of *AtACSs* in response to PBZ treatment in Arabidopsis leaves.**

The third and fourth true leaves from 25-day old plants were detached for PBZ treatment. The samples were collected and subjected to qPCR analysis at indicated time point. *AtACTIN2* was used as an internal control and the expression level of each gene at time 0 was set to 1. Data are means ± SD (*n* = 3 biological replicates). Primers used for qPCR are listed in Table S1.


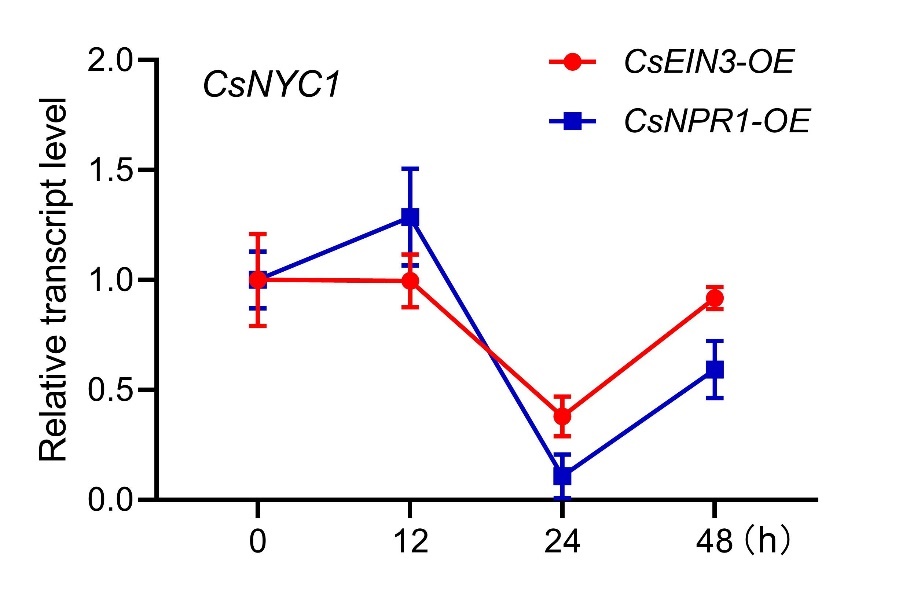


**Figure S4. Time-course expression pattern of *CsNYC1* after overexpression of *CsEIN3* and *CsNPR1* in cucumber cotyledon.**

Relative transcript levels were calculated as the ratio of that in *p35S::CsEIN3*- (or *p35S::CsNPR1*-) vs empty vector-transformed samples. *CsACTIN* (*Csa6G484600*) was used as an internal control and the expression level of *CsNYC1* at time 0 was set to 1. Primers are listed in Table S1. Data are means ± SD (n = 3 biological replicates).


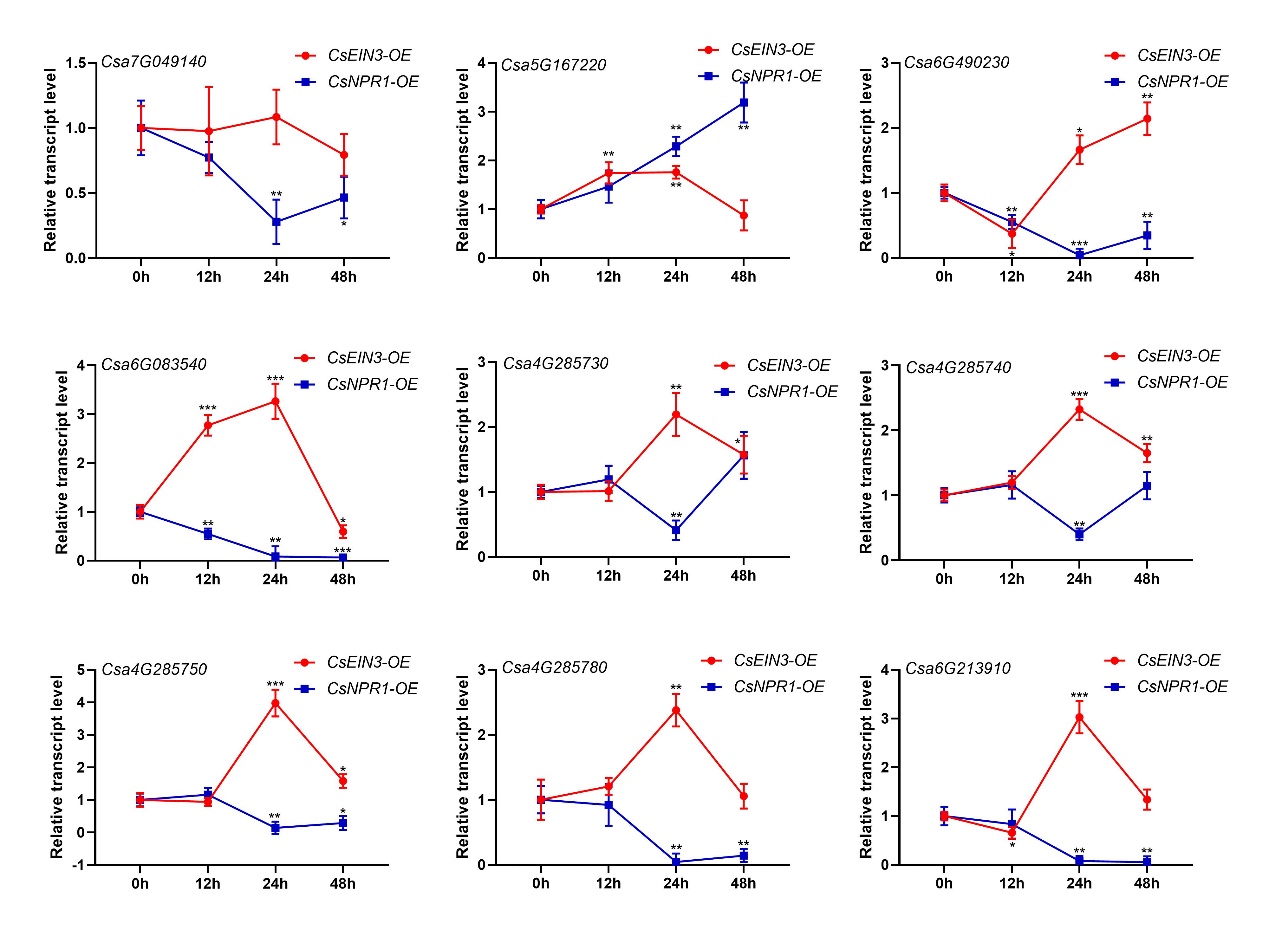


**Figure S5. Time-course expression patterns of *CsPODs* in *CsEIN3*- and *CsNPR1*-overexpressed cucumber cotyledon.**

Relative transcript levels were calculated as the ratio of that in *p35S::CsEIN3*- (or *p35S::CsNPR1*-) vs empty vector-transformed samples. *CsACTIN* (*Csa6G484600*) was used as an internal control and the expression level of each gene at time 0 was set to 1. Gene IDs accorded to the cucumber (Chinese long) v2 genome. Primers are listed in Table S1. Data are means ± SD (n = 3 biological replicates). ****P <* 0.001, ***P <* 0.01, **P <* 0.05 (*t*-test).


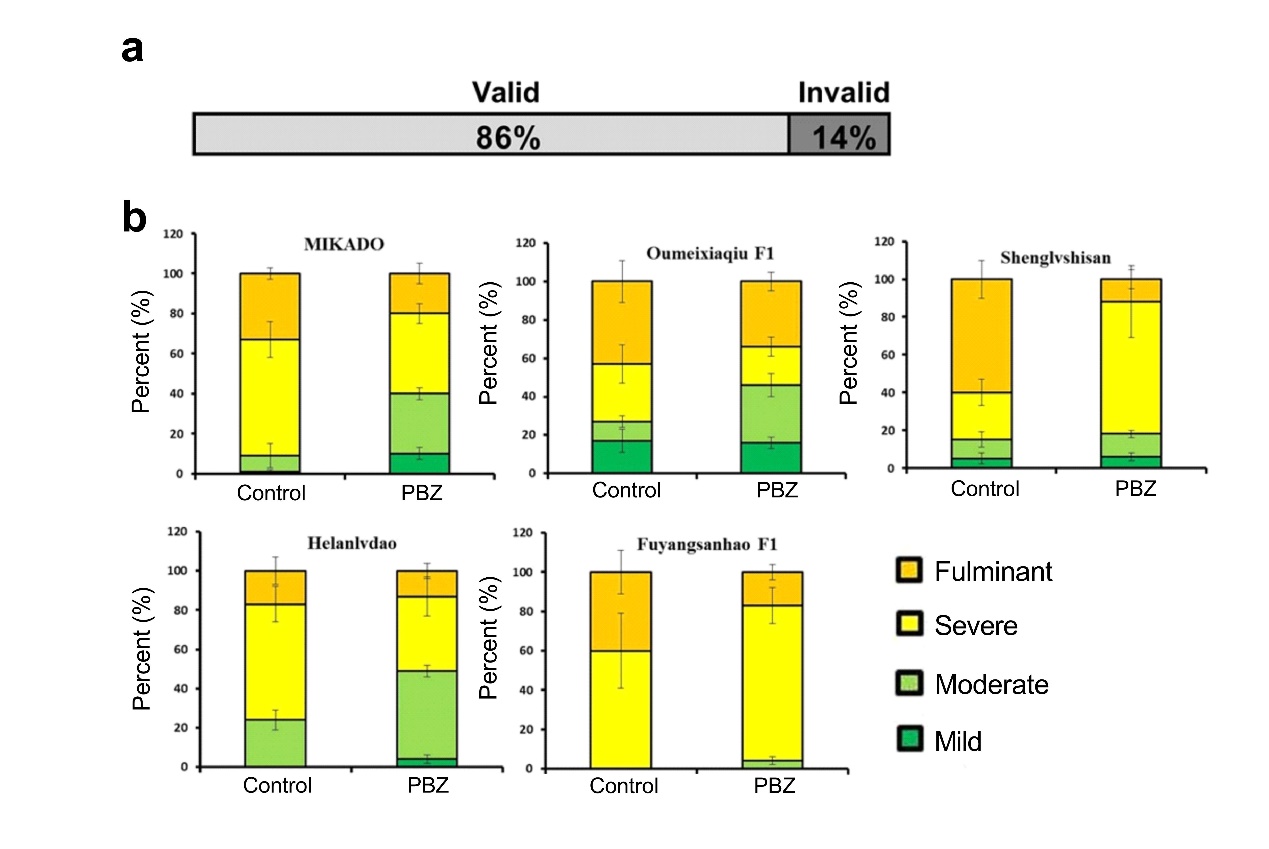


**Figure S6. Test of PBZ inducing powdery mildew resistance in commercial hybrid cucumber cultivars.**

**(a)** Proportion of the valid and invalid cultivars in which PBZ effectively inducing powdery mildew resistance among 36 commercial hybrid cucumber cultivars. **(b)** Statistic data of sick leaves of PBZ-treated cucumber plants in five typical cultivars that PBZ validly induces resistance, with H_2_O treatment used as controls.


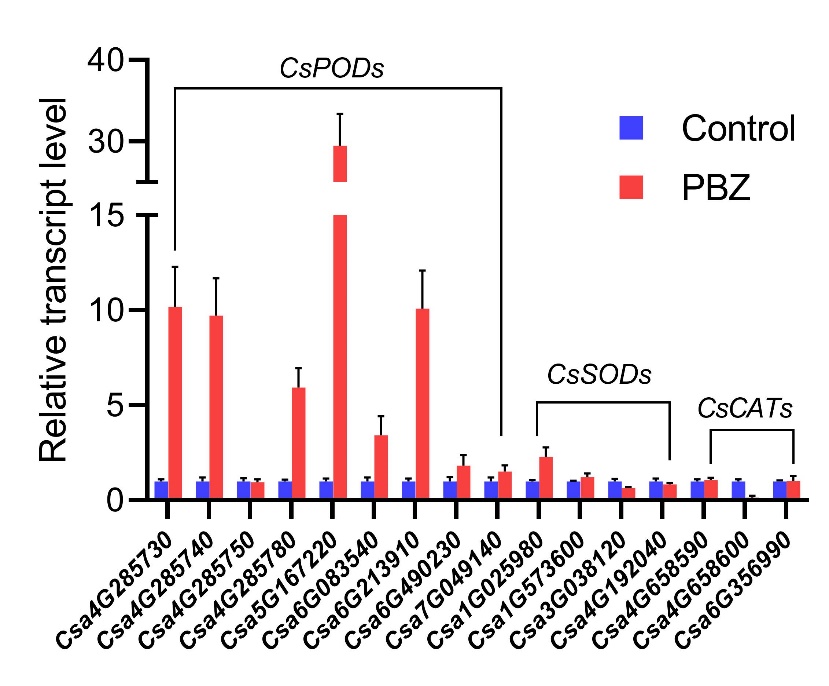


**Figure S7. qPCR analysis the expression of ROS scavenging enzyme genes in PBZ-treated cucumber leaves.**

After 14-day PBZ treatment via root drenching, the first and second true leaves of cucumber plants were used for measurement, with H_2_O treatment as a control. The expression level of each gene in H_2_O treatment was set to 1. Gene IDs accorded to the cucumber (Chinese long) v2 genome. Data are means ± SD (*n* = 3 biological replicates). Primers used for qPCR are listed in Table S1.


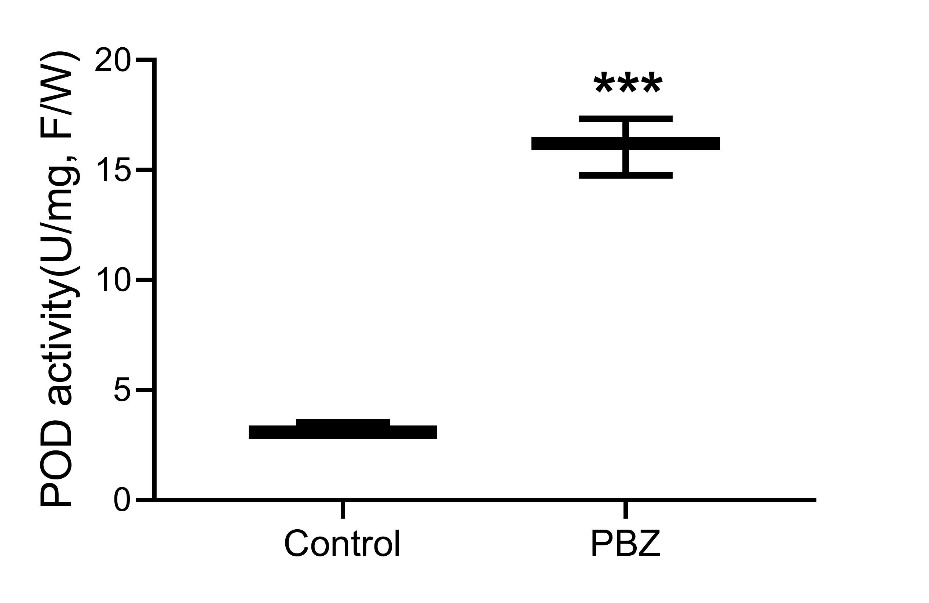


**Figure S8. POD activity in PBZ- and H_2_O- treated cucumber leaves.**

After 14-day PBZ treatment via root drenching, the first and second true leaves of cucumber plants were used for measurement, with H_2_O treatment as a control. Data are means ± SD (*n* = 3 biological replicates). ****P* < 0.001 (*t*-test).


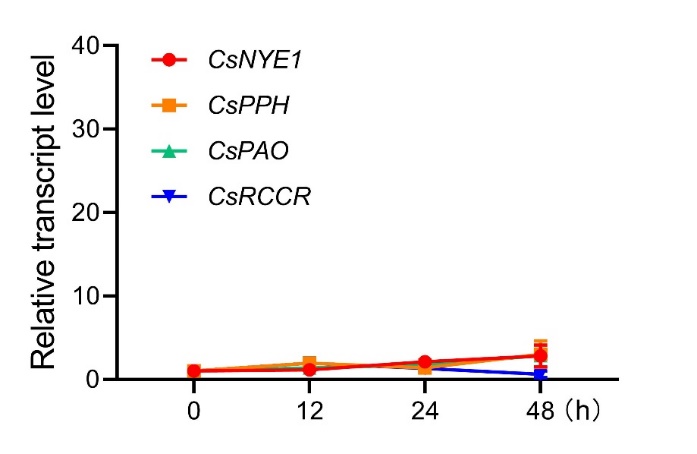


**Figure S9. Time-course expression patterns of *CsCCGs* in response to H_2_O treatment in cucumber leaves.**

Samples are the same as in Figure 5a. Data are means ± SD (*n* = 3 biological replicates).


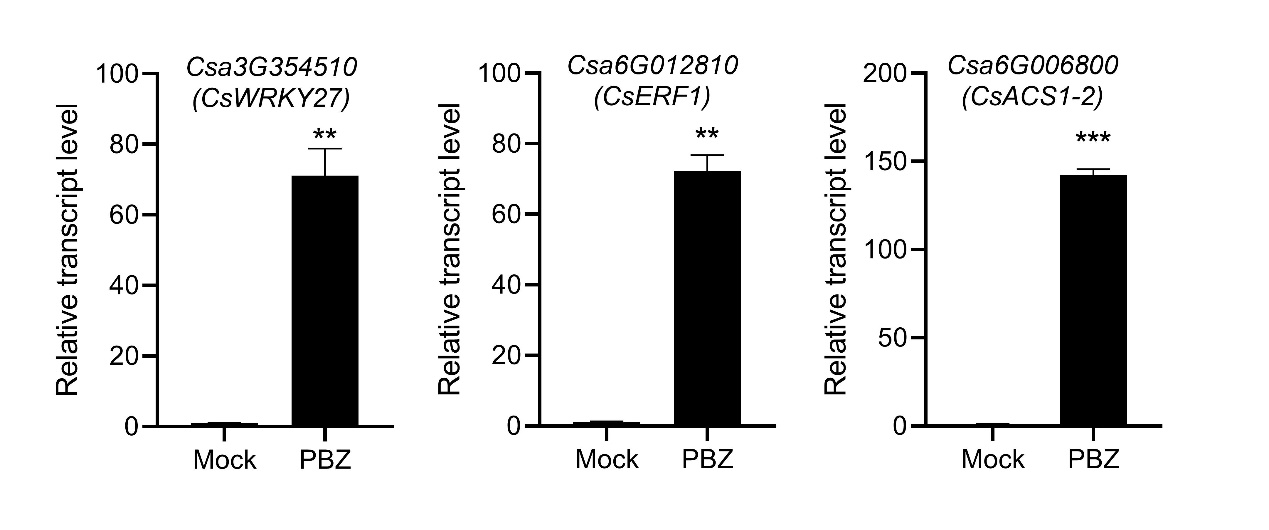


**Figure S10. qPCR validation of three selected DEGs from PBZ-initiated transcriptome versus mock-**

The treatment condition was the same as in Figure 2a. Data are means ± SD (*n* = 2 biological replicates). ***P* < 0.01, ****P* < 0.001 (*t*-test).


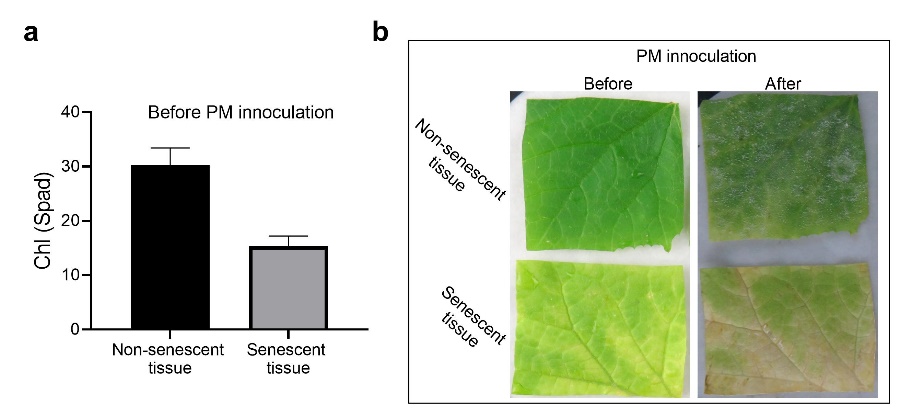


**Figure S11. Leaf senescence inhibits the growth of powdery mildew**

**(a)** Chlorophyll content of cucumber tissues before powdery mildew inoculation. **(b)** Phenotypes of non-senescent and senescent cucumber tissues before and 11 days after powdery mildew inoculation. The experiment was repeated three times with similar results.

**Table S1. Primers used in this work.**

**Table S2. List of DEGs from transcriptome profiling.**
